# Control of breathing by orexinergic signaling in the nucleus tractus solitarii

**DOI:** 10.1038/s41598-024-58075-x

**Published:** 2024-03-29

**Authors:** Yakun Wang, Tianjiao Deng, Xue Zhao, Liuqi Shao, Jinting Chen, Congrui Fu, Wei He, Xiaoyi Wang, Hanqiao Wang, Fang Yuan, Sheng Wang

**Affiliations:** 1 Department of Neurobiology, Hebei Medical University, Shijiazhuang, Hebei China; 2 Department of Sleep Medicine, Third Hospital of Hebei Medical University, Shijiazhuang, China; 3 Nursing School, Hebei Medical University, Shijiazhuang, China; 4 Hebei Key Laboratory of Neurophysiology, Shijiazhuang, China

**Keywords:** Respiration, Neuronal physiology

## Abstract

Orexin signaling plays a facilitatory role in respiration. Abnormalities in orexin levels correlate with disordered breathing patterns and impaired central respiratory chemoreception. Nucleus tractus solitarii (NTS) neurons expressing the transcription factor Phox2b contribute to the chemoreceptive regulation of respiration. However, the extent to which orexinergic signaling modulates respiratory activity in these Phox2b-expressing NTS neurons remains unclear. In the present study, the injection of orexin A into the NTS significantly increased the firing rate of the phrenic nerve. Further analysis using fluorescence in situ hybridization and immunohistochemistry revealed that orexin 1 receptors (OX1Rs) were primarily located in the ventrolateral subdivision of the NTS and expressed in 25% of Phox2b-expressing neurons. Additionally, electrophysiological recordings showed that exposure to orexin A increased the spontaneous firing rate of Phox2b-expressing neurons. Immunostaining experiments with cFos revealed that the OX1R-residing Phox2b-expressing neurons were activated by an 8% CO_2_ stimulus. Crucially, OX1R knockdown in these NTS neurons notably blunted the ventilatory response to 8% CO_2_, alongside an increase in sigh-related apneas. In conclusion, orexinergic signaling in the NTS facilitates breathing through the activation of OX1Rs, which induces the depolarization of Phox2b-expressing neurons. OX1Rs are essential for the involvement of Phox2b-expressing NTS neurons in the hypercapnic ventilatory response.

## Introduction

Sleep-disordered breathing (SDB) is a common and complex condition marked by repetitive episodes of reduced or halted airflow during sleep^1,2^. The primary manifestations of SDB, sleep apnea, and hypoventilation, are influenced by a combination of genetic, anatomical, and neurogenic factors. The dysfunction of respiratory chemoreceptors and the involvement of certain neuropeptides, such as leptin and orexin, are closely linked to these diseases^3–5^.

Orexin, also known as hypocretin, comprises two peptides, orexin A and B, and interacts with two receptors, type 1 and 2 (OX1R and OX2R). These neuropeptides are synthesized by neurons in the lateral hypothalamus (LH) and have a critical role in regulating sleep–wake cycles, energy balance, stress responses, and the reward system^6–8^. Orexinergic neurons project to various brain regions, including key respiratory centers such as the preBötzinger complex (preBötC)^9,10^, the retrotrapezoid nucleus (RTN)^11^ and the nucleus tractus solitarii (NTS)^12^, underpinning its substantial role in respiratory regulation. For instances, activation of the orexin system has been shown to increase respiratory drive^13^. Orexins affect both the inspiratory and expiratory phases of the respiratory cycle, leading to an overall increase in ventilation^14^. Moreover, the orexin system also helps maintain upper airway muscle tone, helping to prevent disturbances such as sleep apnea^15^. Its signaling is implicated in the hypercapnic ventilatory response (HCVR)^16,17^. All these findings suggest that orexinergic signaling contributes to central control of breathing and orexinergic deficiency may result in sleep apnea and hypoventilation.

The NTS is pivotal for the homeostatic regulation of cardiorespiratory functions. Our recent findings demonstrate that stimulation of paired-like homeobox 2b gene (Phox2b)-expressing neurons in the NTS (hereafter called NTS^Phox2b^ neurons) enhances pulmonary ventilation, whereas ablation of this population attenuates the HCVR. Additionally, a subgroup of NTS^Phox2b^ neurons has an intrinsic pH sensitivity^18,19^. Neural tracing data have shown that orexinergic neurons project directly to the NTS^20,21^. The NTS houses both orexin A secreting fibers and neurons expressing OX1Rs^12^. The precise role of orexinergic signaling in influencing the HCVR and its potential excitatory impact on respiration within the NTS, however, has yet to be clarified.

Our current study investigated if orexin A application in the NTS enhances respiratory drive, if OX1R expression coincides with Phox2b, and the necessity of OX1Rs for the HCVR. We demonstrate that orexinergic signaling in the NTS facilitates breathing primarily through increased excitability of NTS^Phox2b^ neurons and plays an important role in central respiratory chemoreception.

## Results

### Injection of orexin A into the NTS potentiates central respiratory drive

**Figure 1:**
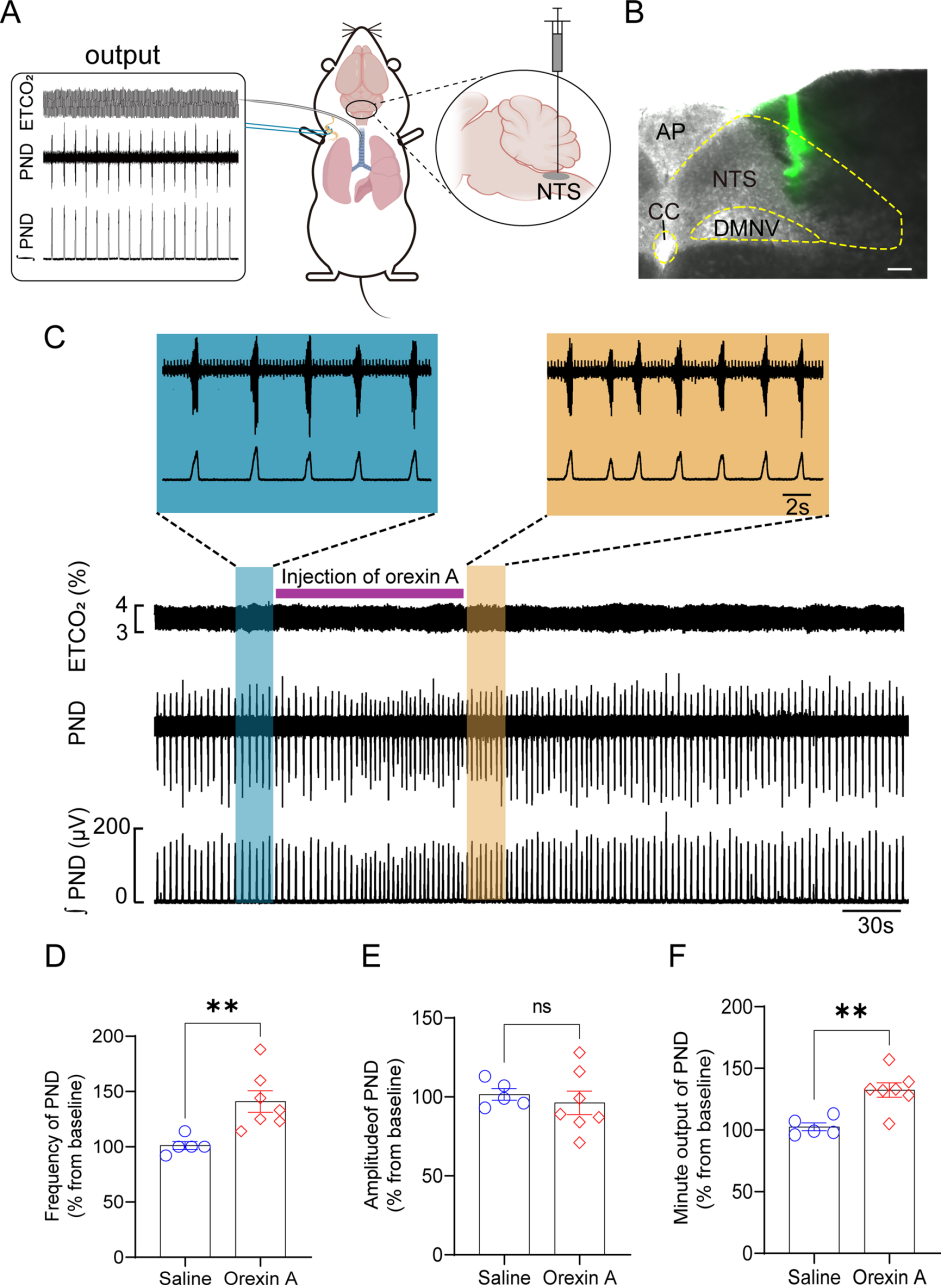
Injection of orexin A in the NTS potentiates PND activity. (**A**) Schematic of the setup showing recordings of PND in anesthetized mice. Top: ETCO_2_ levels; middle: raw PND waveforms; bottom: integrated PND signal, achieved through rectification and smoothing (time constant, 0.05 s). (**B**) Image of the unilateral injection site (green) within the NTS using fluorescent green beads. Scale bar, 50 μm. (**C**) Representative traces showing changes in the PND in response to unilateral injection of orexin A (1 μM, 100 nl per injection) in the NTS. The duration of orexin A injection is marked by a purple line. The blue and orange shadow regions show PND activity measured before and after injection of orexin A, with expanded views shown above for clarity. (**D**–**F**) Quantification of normalized PND frequency, amplitude and minute output in response to injection of either saline or orexin A in the NTS. n = 5 mice for saline, n = 7 mice for orexin A, ^**^P < 0.01 by two-tailed unpaired *t*test. *AP,* area postrema, *CC,* central canal, *DMNV,* dorsal motor nucleus of vagus.

To determine if the activation of orexinergic signaling could enhance central respiratory drive, we delivered orexin A directly into the NTS in anesthetized, bilaterally vagotomized, and mechanically ventilated C57BL/6J mice, while recording phrenic nerve discharge (PND) activity (Fig. 1A). We controlled the end-tidal CO_2_ (ETCO_2_) concentration at a stable 4% to ensure consistent arterial blood gas levels throughout the experiment. To verify the accuracy of each orexin A administration, we co-delivered fluorescent green beads to the same location (Fig. 1B). Tracking of these beads revealed that the injection sites corresponded approximately to the intermediate and ventrolateral subdivisions of the NTS, with an area of dispersion measuring approximately 250–300 µm in the rostrocaudal axis.

When compared to saline injections, unilateral injection of orexin A (1 μM, 100 nL) significantly increased PND frequency (saline: 101 ± 4%; orexin A: 141 ± 10%; n = 5 for saline; n = 7 for orexin A; *P* < 0.01; Fig. 1C, D); however, the change in amplitude was not significant (saline: 102 ± 4%; orexin A: 96 ± 7%; *P* > 0.05; Fig. 1C, E). The stimulatory effect on PND frequency persisted for an average duration of 189 ± 8 s before returning to baseline levels. To quantify the overall respiratory outcome, we determined the minute output of PND (analogous to minute ventilation) by calculating the product of PND frequency and amplitude. As illustrated in Fig. 1F, orexin A administration resulted in a notable elevation in PND output (saline: 103 ± 3%; orexin A: 132 ± 6%; *P* < 0.01). These results consistently suggest that orexin A injection into the NTS markedly enhances the central respiratory drive in anesthetized mice.

### Co-expression of Phox2b and OX1Rs in the NTS

Orexin A is known to elicit its physiological effects predominantly via activating OX1Rs. Building on our recent studies, which have identified the activation of Phox2b-expressing neurons in the NTS as a potent enhancer of respiratory drive, we sought to investigate the potential involvement of orexinergic signaling in this process. To achieve this objective, we employed a combination of RNAscope in situ hybridization and immunohistochemical staining to assess the co-expression of Phox2b and OX1R within the NTS.

**Figure 2:**
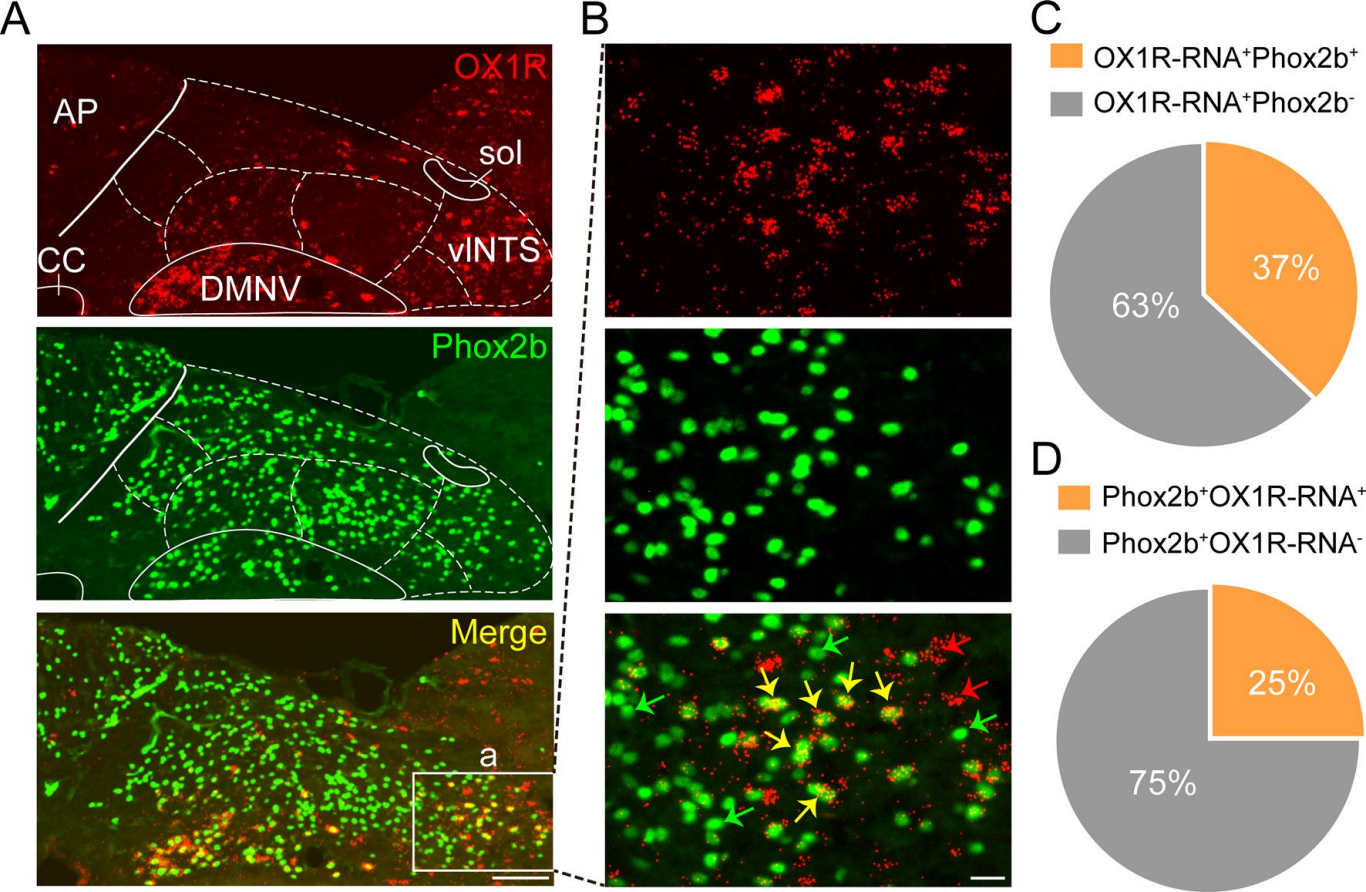
Expression pattern of OX1R-RNA and Phox2b in the NTS. (**A**) OX1R-RNA and Phox2b were identified using RNAscope in situ hybridization and immunohistochemical detection. Images showing the expression of OX1R-RNA (red, top), Phox2b (green, middle) and merged view (bottom) in the NTS. Scale bar, 100 μm. (**B**) Enlarged view of the square in (**A**). The arrows indicate OX1R-RNA^−^Phox2b^+^ (green), OX1R-RNA^+^Phox2b^+^ (yellow), OX1R-RNA^+^Phox2b^−^ (red) neurons, respectively. Scale bar, 20 μm. (**C**,**D**) Quantitative analysis of expression of Phox2b and OX1R-RNA. Cells were manually counted in three sections from each mouse (n = 3 mice). *sol,* solitary tract, *vlNTS,* ventrolateral NTS, *CC,* central canal.

The results, as depicted in Fig. 2A, B, demonstrate that OX1R RNA (shown in red, top) is predominantly present in the ventrolateral segment of the NTS. Alongside, Phox2b protein (shown in green, middle) was found to be abundantly expressed not only in the NTS but also in the area postrema and the dorsal motor nucleus of the vagus. Cell counts were conducted on three sections per mouse across a sample size of three mice. A quantitative analysis revealed that approximately 37% (374 ± 47 per mouse) of OX1R RNA-positive neurons (989 ± 87 per mouse) exhibited immunoreactivity for Phox2b (Fig. 2C). In addition, around 25% (374 ± 47 per mouse) of Phox2b-positive neurons (1522 ± 61 per mouse) in the NTS were found to express OX1R RNA (Fig. 2D). These data provide histomolecular evidence supporting a role for OX1R in the enhancement of respiratory drive, potentially through modulating the activity of Phox2b-expressing neurons in the NTS.

### Orexin A regulates the electrophysiological activity of NTS^Phox2b^ neurons

**Figure 3:**
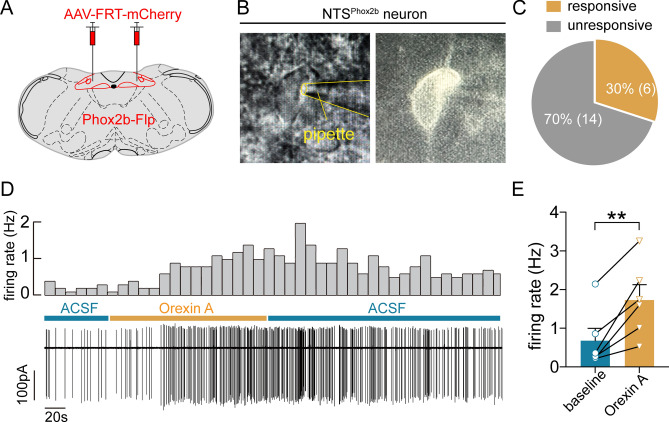
Orexin A regulates electrophysiological activity of NTS^Phox2b^ neurons. (**A**) Schematic of viral injection strategy in Phox2b-Flp mice. (**B**) Images showing a fluorescent NTS^Phox2b^ neurons in a brain slice using infrared differential interference contrast optics (left) and fluorescence microscopy (right). (**C**) Proportion of responsive (30%) and unresponsive (70%) NTS^Phox2b^ neurons. n = 20 neurons from four mice. (**D**) Firing activity of a fluorescent NTS^Phox2b^ neuron during bath application of orexin A (100 nM). Firing rate histograms (top traces; bin size, 10 s) were derived from cell-attached voltage-clamp recordings (bottom traces). (**E**) Quantitative analysis of firing rate in responsive NTS^Phox2b^ neurons (n = 6, ^**^P < 0.01, two-tailed paired *t* test).

To elucidate the role of orexin A in modulating the electrophysiological properties of NTS^Phox2b^ neurons, we injected a Flp/FRT-inducible virus into the NTS of Phox2b-Flp mice 4 weeks in advance of our electrophysiological experiments (Fig. 3A). For recording purposes, we utilized cell-attached patch-clamp techniques to capture the spontaneous firing activity of fluorescently-marked NTS^Phox2b^ neurons in brain slices, focusing on cells within the intermediate and ventrolateral subdivisions of the NTS (Fig. 3B). Our recordings identified twenty NTS^Phox2b^ neurons that exhibited spontaneous activity (Fig. 3C). Following bath application of orexin A (100 nM), a substantial increase in firing rate was observed in 6 out of these 20 neurons (0.7 ± 0.3 Hz vs*.* 1.7 ± 0.4 Hz, basal vs*.* post-orexin A application; *P* < 0.01; Fig. 3D, E). The remaining 14 neurons displayed no discernible response to orexin A administration. Drawing on these observations, we postulate that the excitatory influence on this specific subset of NTS^Phox2b^ neurons may be attributed to OX1R activation. These insights augment our comprehension of the selective impact of orexin A on neuronal electrophysiology within the NTS.

### CO_2_ activation of OX1R RNA-expressing NTS^Phox2b^ neurons

**Figure 4:**
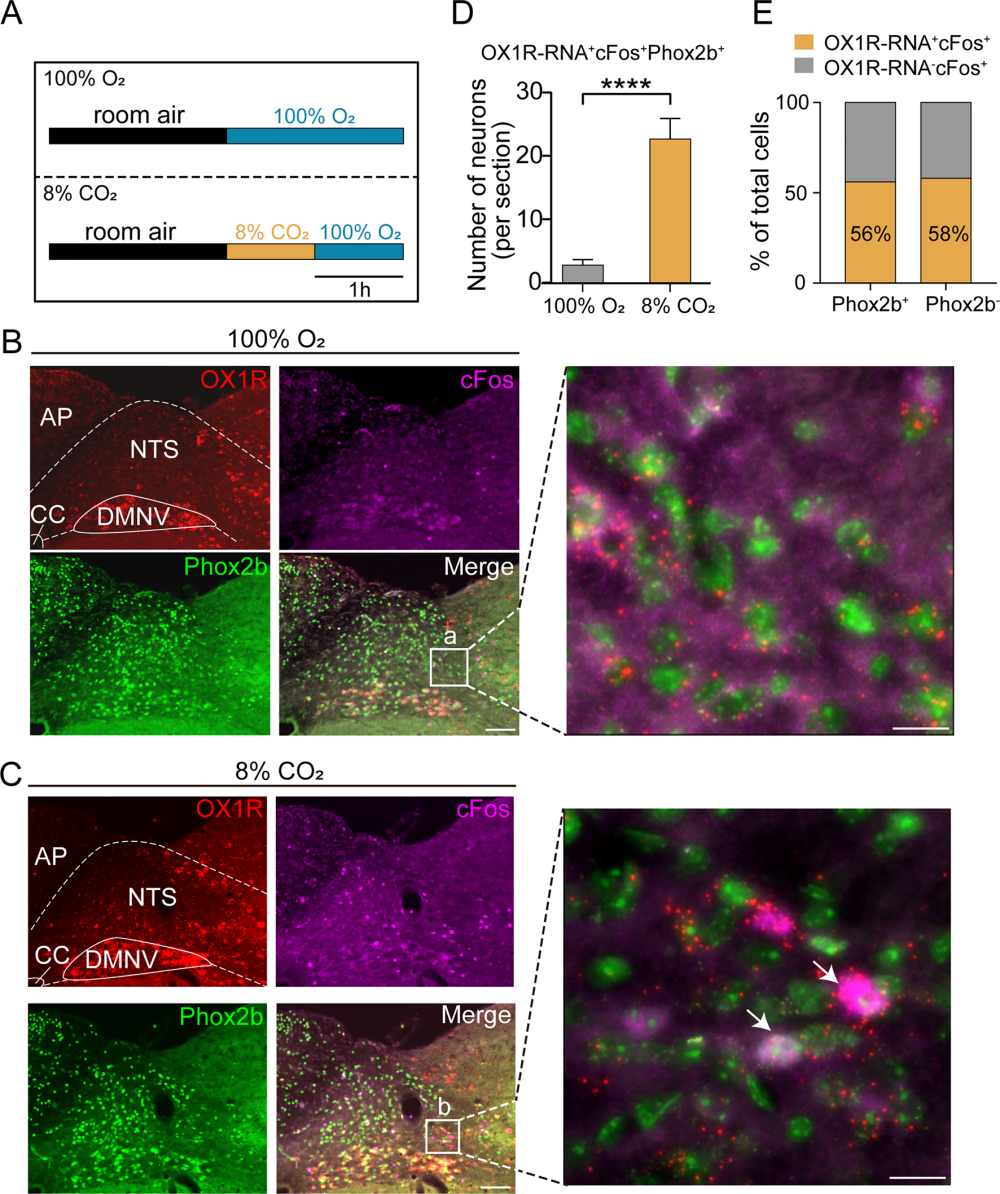
Identification of CO_2_-activated OX1R-RNA-residing NTS^Phox2b^ neurons. (**A**) Schematic diagram illustrating experimental protocols before histological experiments. (**B**,**C**) Combined application of RNAscope in situ hybridization and immunofluorescence staining showed co-expression of OX1R-RNA^+^ (red), cFos^+^ (purple) and Phox2b^+^ (green) neurons in the NTS. CO_2_-activated neurons were indicated by immunoreactivity to cFos. Square area-indicated views were enlarged in the right images. The arrows indicate co-expressing OX1R-RNA, cFos and Phox2b in NTS neurons. Scale bars, 100 μm (**B**,**C**, left), 20 µm (**B**,**C**, right). (**D**) Quantification of the number of OX1R-RNA^+^cFos^+^Phox2b^+^ neurons from mice exposing to either 100% O_2_ and 8% CO_2_. Cells were counted from seven sections (n = 3 mice) for 100% O_2_ and eight sections (n = 3 mice) for 8% CO_2_. ^****^P < 0.0001, 100% O_2_ vs*.* 8% CO_2_, unpaired *t* test. (**E**) Cell count analysis showing that 56% of CO_2_-activated NTS^Phox2b^ neurons and 58% of CO_2_-activated Phox2b^−^ neurons expressed OX1R-RNA.

A certain subgroup of NTS^Phox2b^ neurons has been previously identified as sensitive to increased levels of CO_2_/H^+^, functioning as central respiratory chemoreceptors^18^. Building on this understanding, we sought to determine if NTS^Phox2b^ neurons harboring the OX1Rs could be activated by elevated CO_2_ levels. To explore this, we exposed C57BL/6 J mice to atmospheres containing either 100% O_2_ or 8% CO_2_ (Fig. 4A). Subsequently, we performed RNAscope in situ hybridization alongside immunohistochemical staining to detect the expression of cFos, OX1R RNA, and Phox2b within NTS neurons.

The number of cells was counted in seven sections from three mice treated with 100% O_2_, and eight sections from three mice exposing to 8% CO_2_. Quantitative analysis demonstrated a greater increase in OX1R-RNA^+^cFos^+^Phox2b^+^ neurons from mice exposing to 8% CO_2_ relative to 100% O_2_ (3 ± 1 vs*.* 23 ± 3 per section, 100% O_2_ vs*.* 8% CO_2_; *P* < 0.0001; Fig. 4B–D). In addition, 56% of cFos^+^Phox2b^+^ neurons (40 ± 2 per section) were OX1R-RNA^+^ (23 ± 3 per section, Fig. 4E). Moreover, 58% of cFos^+^Phox2b^-^ neurons (57 ± 3 per section) were OX1R-RNA^+^(32 ± 1 per section, Fig. 4E). This evidence indicates that CO_2_ can activate an OX1R-positive subset of NTS^Phox2b^ neurons, supporting the concept of a multifaceted chemoreceptive role for orexin signaling in the central respiratory network.

### Knockdown of OX1Rs in NTS^Phox2b^ neurons attenuates the HCVR

**Figure 5:**
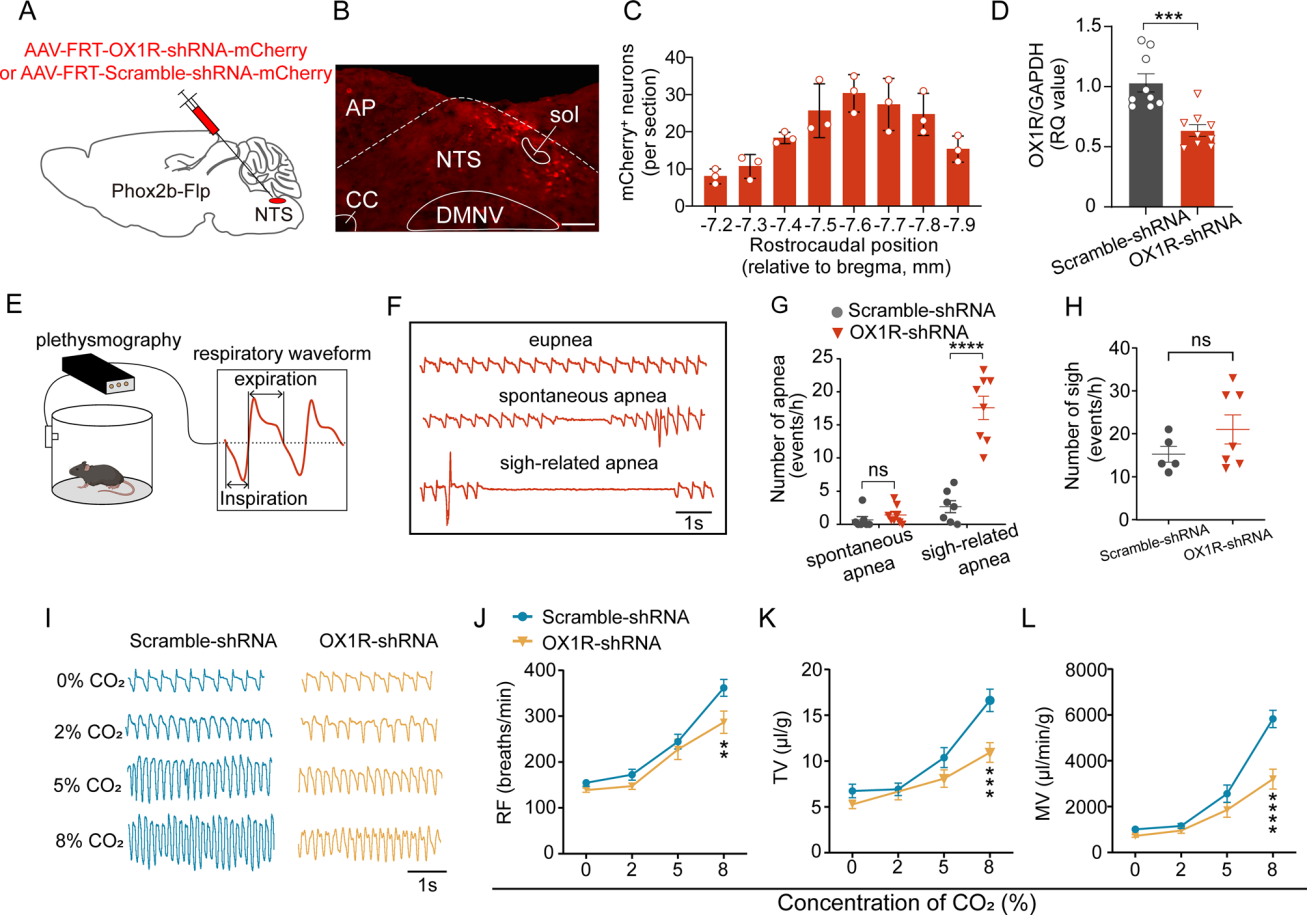
Knockdown of OX1R-RNA in NTS^Phox2b^ neurons blunted the HCVR. (**A**) Schematic diagram illustrating a genetic strategy to knockdown OX1R-RNA through bilateral injections of the FRT-inducible virus AAV-FRT-OX1R-shRNA-mCherry (AAV-FRT-Scramble-shRNA-mCherry as control group) into the NTS from Phox2b-Flp mice. (**B**) Immunohistochemical validation of mCherry-expressing neurons. Scale bar, 200 µm. (**C**) Rostrocaudal distribution of mCherry-expressing neurons. Cells were counted in eight coronal sections (bregma: − 7.2 to − 7.9 mm; thickness: 25 µm; each separated by 75 µm) from each mouse (n = 3 mice). (**D**) qPCR to verify knockdown effectiveness. Following knockdown, ~ 64% of OX1R-RNA in NTS^Phox2b^ neurons was retained (^***^P < 0.001, three technical replicates for each of three mice for both groups, two-tailed unpaired *t* test). (**E**) Schematic of monitoring respiratory function with the whole body plethysmography. (**F**) Typical traces showing eupnea, spontaneous apnea and sigh-related apnea. (**G**) Knockdown of OX1R-RNA in NTS^Phox2b^ neurons increases sigh-related apnea, rather than spontaneous apnea (n = 7 mice for scramble group, n = 8 mice for knockdown group, ^****^P < 0.0001, two-tailed unpaired *t* test). (**H**) Knockdown of OX1R-RNA in NTS^Phox2b^ neurons caused no significant change in the number of sighs. n = 5 mice for scramble group, n = 7 mice for knockdown group. Two-tailed unpaired *t* test. (**I**) The original airflow waveforms illustrating the HCVR in both Scramble-shRNA and OX1R-shRNA groups. (**J**–**L**) Effect of knockdown of OX1R-RNA in NTS^Phox2b^ neurons on the HCVR. Knockdown of OX1R-RNA in NTS^Phox2b^ neurons caused an insignificant change in baseline breathing parameters but significantly blunted the HCVR during exposure to 8% CO_2_ (n = 9 mice for each group, ^**^P < 0.01, ^***^P < 0.001, ^****^P < 0.0001, two-way ANOVA with Sidak’s multiple comparisons test). *ns,* not significant.

Upon establishing that a subset of NTS^Phox2b^ neurons containing OX1R RNA could be activated by CO_2_, we aimed to assess whether these neurons are essential for the HCVR. To this end, we bilaterally injected FRT-inducible viruses encoding OX1R-shRNA into the NTS of Phox2b-Flp mice (Fig. 5A), followed by immunohistochemical validation of the distribution of transduced neurons (Fig. 5B, C). The quantitative PCR data indicated the retention of approximately 64% of OX1R mRNA in the NTS^Phox2b^ neurons of mice injected with OX1R-shRNA compared to those injected with scramble-shRNA (*P* < 0.001, with three technical replicates for each of three mice from both groups; Fig. 5D).

Subsequently, we assessed the ventilatory response using whole body plethysmography (WBP) in behaviorally quiescent mice (Fig. 5E). Initially, we noted a higher occurrence of sigh-related apneas in the OX1R-shRNA group compared to the scramble-shRNA group (3 ± 1 events/h vs*.* 18 ± 2 events/h, scramble-shRNA vs*.* OX1R-shRNA; n = 7 for scramble-shRNA, n = 8 for OX1R-shRNA, *P* < 0.0001; Fig. 5F, G), while the frequency of spontaneous apneas did not differ between both groups (Fig. 5F, G). Furthermore, we calculated the number of sighs based on 3 h WBP recordings. The average of sighs was comparable between the two groups of mice (Fig. 5H).

During exposure to various CO_2_ levels, we methodically measured respiratory frequency (RF), tidal volume (TV), and minute ventilation (MV) across both groups. While breathing 100% O_2_, alongside 2% and 5% CO_2_, no statistically significant changes in breathing parameters were observed between the two groups. However, when challenged with 8% CO_2_, the OX1R-shRNA group demonstrated a notable decrease in RF, TV, and MV compared to the scramble-shRNA group (RF: 362 ± 18 breaths/min vs*.* 287 ± 24 breaths/min, *P* < 0.01; TV: 17 ± 1 μl/g vs*.* 11 ± 1 μl/g, *P* < 0.001; MV: 5828 ± 377 μl/min/g vs*.* 3205 ± 433 μl/min/g, *P* < 0.0001; scramble-shRNA group vs*.* OX1R-shRNA group; n = 9 mice per group; Fig. 5I–L). These findings suggest that knockdown of OX1Rs in NTS^Phox2b^ neurons is associated with an increased likelihood of apnea and a diminished HCVR.

## Discussion

In the present study, we present evidence that administering orexin A directly into the NTS augments central respiratory drive in mice. The enhancement of respiratory function appears to be primarily due to the activation of OX1R-residing NTS^Phox2b^ neurons, which most likely provides excitatory input to the ventral respiratory group. Furthermore, we observed that the targeted knockdown of OX1Rs on these NTS^Phox2b^ neurons led to a diminished HCVR, alongside with an increased incidence of apnea episodes with clear pathological characteristics.

Our previous studies have demonstrated that activation of NTS^Phox2b^ neurons plays a significant role in promoting pulmonary ventilation. Despite their function as central respiratory nodes, the activity of these neurons is modulated by several intrinsic factors, including ion channels and G protein-coupled receptors on their membranes, as well as by excitatory and inhibitory synaptic inputs ranging from the brainstem to the cortex, in addition to paracrine signaling mechanisms. Building on this foundation, we have reported that leptin signaling within the NTS enhances respiration under normal physiological conditions via an NTS-lateral parabrachial nucleus circuit^22^. Moreover, disrupted leptin signaling in the NTS and RTN has been closely linked with hypoventilation and a compromised HCVR in an obese mouse model^23,24^. Similarly, the contribution of orexinergic signaling to respiratory control is an area requiring further investigation.

Orexinergic fibers target multiple respiratory-related nuclei, such as the preBötC^9,10^, Kölliker–Fuse nucleus^10,25,26^, RTN^11^, NTS^12,27^ and locus coeruleus^28^. Several studies have shown that orexins can mediate respiratory chemoreflex responses and augment TV^29–31^. For instance, microinjecting orexin into the preBötC or the phrenic motor nucleus increased diaphragm activity in adult rats in vivo^29^, and applying orexin-B in the Kölliker–Fuse nucleus raised the baseline respiratory frequency^32^. Additionally, Fukushi et al. also found that expression of OX2R was identified in the parafacial respiratory group/RTN and the preBötC, and a dual orexin receptor antagonist suppressed the amplified HCVR in adult mice^33^. While these data have illuminated the regulatory role of orexinergic signaling in the preBötC, RTN and Kölliker–Fuse nucleus, knowledge on how this peptide affects breathing via the NTS has remained elusive. Our previous findings showed that stimulation of NTS^Phox2b^ neurons potentiate ventilation, presenting a plausible central intervention point for SDB conditions like congenital central hypoventilation syndrome, which is characterized by mutations in Phox2b within affected individuals. In this current study, we unveiled that application of orexin A to the NTS intensified the excitatory impetus for respiration. Notably, OX1Rs were identified on a subset of NTS^Phox2b^ neurons, particularly within the ventrolateral subdivision of NTS. Consequently, the joint activation of both the orexin and leptin systems in the NTS has been shown to facilitate breathing in mice, reinforcing the potential of orexinergic signaling as a target in respiratory regulation and offering insights for the clinical management of respiratory conditions. However, due to the expression of OX1Rs and OX2Rs in astrocytes as reported previously^34,35^, the present study cannot rule out the contribution of astrocytic activation in the NTS to Orexin A-induced enhancement of respiratory drive.

Accumulated evidence has reported that OX1R activation can increase the frequency of spontaneous excitatory postsynaptic currents and evoke excitatory synaptic transmission within the NTS^36^. Complementing this, Yang and colleagues found that orexin A induces depolarization in NTS neurons^37^. Correlating with these findings, our work has further revealed that exposure to orexin A leads to depolarization of NTS^Phox2b^ neurons. Although we pharmacologically blocked glutamatergic, GABAergic and glycinergic transmission, it is possible that orexin A acts on its receptors located in astrocytes in the NTS based on the existing data^34,35^, ultimately resulting in such a depolarization. Synthesizing the insights from prior research with our present observations, we hypothesize that orexin A acts upon OX1Rs within NTS^Phox2b^ neurons, amplifying their excitability and thereby facilitating the initiation of respiratory activity.

The HCVR is crucial for maintaining arterial CO_2_ homeostasis. A substantial body of evidence has uncovered the involvement of the orexin system in the HCVR^38^. For example, transgenic mice lacking orexin display a diminished ventilatory response to elevated CO_2_ or hypercapnic chemoreflex^39^. Moreover, the administration of orexin A or B has been demonstrated to partially rectify the dysfunctional CO_2_ chemoreflex in orexin-deficient mice^31^. The pharmacological inhibition of orexin receptors induces a phenotype akin to that observed in orexin-deficient mice^31,40^. Li et al. reported that oral administration of the dual orexin receptor antagonist almorexant significantly reduced the ventilatory response to hypercapnia (7% inhaled CO_2_) by 26%, and this effect was confined to the wakeful, active phase of the circadian cycle^40^. Similarly, Deng et al. also observed that intracerebroventricular administration of orexin receptor antagonist (SB-334867) produced a similarly attenuated ventilatory response to CO_2_ in wakefulness^31^. However, the contribution of orexinergic signaling within the NTS to the HCVR is not well understood. Our current study revealed that OX1R-expressing NTS^Phox2b^ neurons are activated when inhaling 8% CO_2_. Additionally, we demonstrated that the down-regulation of OX1Rs on NTS^Phox2b^ neurons resulted in a remarkable attenuation of the HCVR in response to 8% CO_2_ exposure. Since orexin is released from orexin-expressing LH neurons, we suggest that these neurons exert a facilitatory effect on ventilation through acting on downstream NTS^Phox2b^ neurons. However, the specific cellular mechanisms underlying the impact of OX1R activation on the CO_2_/H^+^ sensitivity of NTS^Phox2b^ neurons require further elucidation.

Our findings also suggest an increased frequency of sigh-related apnea following the reduction of OX1Rs in NTS^Phox2b^ neurons. Consistent with our observations, orexin knockout mice have exhibited not only an increase in the number of apneas but also a blunted HCVR while maintaining near-normal resting ventilation^41^. Meanwhile, our data demonstrate that knockdown of OX1Rs in NTS^Phox2b^ neurons caused no significant change in the number of sighs. Recent study reveals that optogenetic stimulation of orexin-expressing LH neurons induces sighing through activation of the RTN-preBötC circuit^42^. However, it is still possible that stimulation of orexin-expressing LH neurons projecting to the NTS evokes sighing. Whether the LH-NTS-preBötC circuit mediates regulation of sighing remains unknown and awaits to be further revealed. In clinical observations, narcolepsy patients with a deficiency in hypocretin-1 suffer from more frequent and severe sleep apnea episodes^43–45^. Therefore, additional clinical and basic research are required to clarify the relationship between orexinergic signaling and apnea with the aim of exploring its potential therapeutic value in treating SDB.

In summary, orexinergic signaling in the NTS facilitates respiratory activity by activating OX1Rs, which in turn, trigger the depolarization of NTS^Phox2b^ neurons. The engagement of this population of neurons in the ventilatory response to hypercapnia is to some extent dependent on the function of OX1Rs. These findings provide potential therapeutic target for improvement of SDB.

## Methods

### Animals

The animal used in this experiment consisted of 8–12 weeks old C57BL/6J and Phox2b-Flp mice of either sex. Phox2b-Flp mice were obtained from Jackson Labs (stock number: 022407) and were validated previously^46,47^. The Phox2b-Flp mice were genetically modified to express both Phox2b and Flp recombinase, through the insertion of the Flp recombinase sequence into the Phox2b exon. The genetic modification allows for specific targeting and manipulation of Phox2b neurons by the Flp recombinase, which possesses the ability to selectively recognize viruses containing FRT sequence. All mice were housed on a fixed 12 h light/12 h dark cycle, with ad libitum access to food and water. All experiments were carried out in conformity to the Guide for the Care and Use of Laboratory Animals, and were approved by Animal Care and Ethics Committee of Hebei Medical University. In addition, all methods were performed in accordance with the relevant guidelines and regulations.

### Viral vectors and surgery

The viral vectors involved in this study include AAV-FRT-Scramble-shRNA-mCherry, AAV-FRT-OX1R-shRNA-mCherry and AAV-FRT-mCherry, all of which were stored at a temperature of − 80 ℃ in a freezer. The surgical procedures were performed under aseptic conditions. The mice were anesthetized by pentobarbital sodium (60 μg/g, i.p.), with additional anesthesia administered if necessary (30% of the original dose). The depth of anesthesia was determined by the absence of corneal reflexes and digital clipping reflexes. The mice were fixed on the stereotaxic apparatus (RWD, China) in a prone position, while body temperature was maintained at 37 °C by a heating pad, and the eyes were protected by ophthalmic ointment.

In the context of NTS injection, viruses or drugs were injected into the middle and caudal regions as needed (stereotaxic coordinates: anterior/posterior 0.2 mm, medial/lateral ± 0.3 mm, dorsal/ventral − 0.1 mm; and anterior/posterior 0.4 mm, medial/lateral ± 0.4 mm, dorsal/ventral − 0.2 mm, with the calamus scriptorius as the zero coordinate). All coordinates refer to the mouse brain in stereotaxic coordinates^48^. Viruses or drugs were injected at a rate of 50 nL/min, with a volume of 100 nl per site, and the glass microelectrode remains in situ for at least 5 min to allow the virus to diffuse completely. After the operation, the mice were intraperitoneally injected with ampicillin (125 mg/kg) and ibuprofen (4 mg/kg), and recovered for 4 weeks before the subsequent experiment.

### PND recording

The protocol was used as described previously^22^. Mice were anesthetized by urethane (1.3 g/kg body weight, i.p.). Additional doses of 0.1 g/kg could be appropriately supplemented as needed. Bilateral vagus nerve dissociation was performed after tracheal intubation of the mice. Then, Pancuronium (5 mg/kg body weight, i.p.), a muscle relaxant, was administered. The mice were immediately accepted mechanical ventilation (ventilator: SAR-1000, CWE, USA) with 100% O_2_ in order to inactivate peripheral chemoreceptors. Meanwhile, ETCO_2_ level was continually monitored with a capnograph (MicroCapStar, CWE Inc., USA) and sustained at approximately 4% as the baseline level. The left phrenic nerve was isolated under a microscope and suspended on a silver bipolar electrode, then submerged in warm paraffin oil. All analog data were amplified via a micro1401 digitizer (Cambridge Electronic Design Ltd, UK) and subsequently analyzed with Spike 2 software (RRID: SCR-000903, Cambridge Electronic Design). The original PND was sampled at a rate of 2 kHz and filtered with a bandpass of 30–3000 Hz. The integrated PND was obtained after rectification and smoothing (time constant, 0.05 s) of the original signal. The frequency and peak amplitude of the integrated PND were used for statistical analysis.

### Electrophysiological recordings in brainstem slices

Transverse brainstem slices were prepared from Phox2b-Flp transgenic mice 4 weeks after injection of AAV-FRT-mCherry in the NTS as previously described^18^. Following decapitation under deep anesthesia (5% pentobarbital sodium at a dose of 1.5 ml/kg), the brainstem was removed and coronal slices (250 μm, thickness) were cut with a vibratome (VT1200S; Leica Biosystems, Wetzlar, Germany) in an ice-cold sucrose-containing solution (in mM: 260 sucrose, 3 KCl, 2 MgCl_2_, 2CaCl_2_, 1.25NaH_2_PO_4_, 26 NaHCO_3_, 1 glucose and 1 kynurenic acid, saturated with 95% O_2_-5% CO_2_, pH 7.4, 300 mOsm). Before recording, slices were incubated for 1 h in solution containing the ingredients (in mM): 140 NaCl, 3 KCl, 2 MgCl_2_, 2 CaCl_2_, 10 Hepes and 10 glucoses, pH adjusted to 7.4 by adding HCl and NaOH.

Cell-attached patch clamp recordings were performed in mCherry-labeled neurons in brainstem slices at room temperature using pClamp, a Multiclamp 700B amplifier and a Digidata 1440A analog-to-digital converter (all from Molecular Devices, Sunnyvale, CA, USA). The recordings were conducted in a bath solution that was superfused continuously at a speed of 2 ml/min. The patch pipettes (3–6 MΩ) were filled with (in mM): 120 KCH_3_SO_3_, 4 NaCl, 1 MgCl_2_, 0.5 CaCl_2_, 10 HEPES, 10 EGTA, 3 Mg-ATP and 0.3 GTP-Tris, pH adjusted to 7.2 with KOH. Cell-attached recordings were performed at a holding potential of − 60 mV. All electrophysiological recordings were made in the presence of picrotoxin (50 μM) and 6-cyano-7-nitroquinoxaline-2,3-dione (10 μM) and strychnine (30 μM) to block fast excitatory and inhibitory synaptic transmission. Firing rate histograms were constructed by integrating the discharge of action potentials in 10 s bins using Spike 2 software (Cambridge Electronic Design, Cambridge, UK). Orexin A was purchased from Tocris Bioscience (Shanghai, China).

### Respiratory monitoring

In this study, the evaluation of HCVR and apnea in freely-moving mice was conducted via WBP (EMKA Technologies, Paris, France) as depicted before^19^. A mass flow regulator provided quiet, constant and smooth flow through the animal chamber (0.5 l/min). Mice were adapted in the plethysmography chamber to allow for acclimatization period 3–4 h prior to testing and 2 h before the testing protocol.

The HCVR protocol was carried out as follows: a series of four CO_2_ challenges were administered sequentially, with each challenge consisting of a 7 min exposure to CO_2_ concentrations of 0%, 2%, 5%, and 8%, balanced with O_2_, and separated by 5 min of exposure to 100% O_2_. The hypercapnic exposure was performed in hyperoxia to minimize the influence of peripheral chemoreceptors on the hypercapnic ventilatory reflex and attribute the observed ventilatory effects to central chemoreception. The CO_2_ level in the chambers was verified with a capnograph. Ventilatory flow signal was recorded, amplified, digitized and analyzed using IOX 2.7 (EMKA Technologies) to ascertain ventilatory parameters over sequential 20 s epochs (~ 50 breaths), during periods of behavioral quiescence and regular breathing. MV (μl/min/g) was calculated as the product of the RF (breaths/min) and TV (μl/g), normalized to the body weight of mouse (g).

A 3 h recording of quiescent normoxic breathing signal was conducted for the purpose of apnea analysis. Raw respiratory waveform data were processed and analyzed using Spike 2 software (Cambridge Electronic Design). The apneas identified by the software were subsequently verified manually. According to previous investigations^49^, spontaneous apneas were operationally defined as breathing intervals with an expiratory time exceeding 1.2 s, equivalent to approximately 3 normal breath cycles for C57BL/6J mice with a breathing frequency of 150 breaths/min, or ~ 400 ms/breath, and not preceded by a sigh. Considering the distinct central mechanisms, we distinguish these spontaneous from sigh-related apneas. Sighs were defined as a substantial increase in inhalation followed by deep expiration, while apneas were regarded as sigh-associated if they occurred within 10 breaths of the sigh. In addition, to exclude the influence of orexin fluctuation with day/night cycle, all the WBP recording, together with the above PND recordings, were made at the same time (8am to 2 pm) of each experimental day.

For cFos-based histological analysis of CO_2_-activated neurons in vivo, mice were accustomed to the plethysmography chamber for 4–6 h 1 day in advance, and again for 2 h prior to the protocol. Subsequently, the mice were then exposed to 8% CO_2_ stimulus (hyperoxic hypercapnia) for 1 h, followed by 100% O_2_ for 1 h, and mice exposure to 100% O_2_ for 2 h were considered as control. Afterwards, mice were instantly anesthetized and perfused transcardially with fixative for subsequent histological processing.

### Histology and RNAscope in situ hybridization

The detection of cFos, Phox2b and OX1R mRNA transcripts were performed simultaneously by utilizing the RNAscope in situ hybridization and immunohistochemistry. In brief, mice were fixed with paraformaldehyde (PFA, 4%, 4 ℃) after anaesthetization, and brain tissue was collected and immersed in PFA (4 ℃) for 24 h. Subsequently, the tissue was dehydrated through a gradient of 15% and 30% sucrose phosphate buffer (PB) solution. After that, the tissue was embedded in OCT and stored at − 80 ℃. Coronal sections (15 μm) were obtained using a cryostat (CM1950; Leica Microsystems, Wetzlar, Germany). According to the manufacturer’s user manual for the RNAscope^®^ multichannel second-generation fluorescence kit (document no. 323100-USM), the slides were subjected to the RNAscope multiplex fluorescent assay. Subsequently, images were acquired via a confocal microscope (LSM 800; Carl Zeiss, Jena, Germany) and processed with the ZEN software program (Carl Zeiss).

### qPCR

For loss-of-function experiments, qPCR was applied to verify the knockdown effectiveness of OX1Rs. Total RNA was extracted utilizing the Eastep^®^Super Total RNA Extraction Kit (Shanghai Promega, Cat.# LS1040). The HiScript^®^ III RT SuperMix for qPCR (+gDNA wiper) (vazyme #R323) was used for cDNA synthesis. The mRNA expression level was analyzed by real-time quantitative PCR instrument (Quant Studio 6 Flex, applied biosystems, USA) with ChamQ SYBR qPCR master mix (without ROX) (vazyme #R321). The primers’ sequences were shown as follows: forward, CTGTGGCGCGATTATCTCTAC, and reverse, GCCAGGGACAGGTTGACAA for OX1R (hypocretin/orexin receptor 1; amplicon: 284 bp, Gene ID: 230777); forward, AAATGGTGAAGGTCGGTGTGAACG, and reverse, AGTGATGGCATGGACTGTGGTCAT for GAPDH (Glyceraldehyde 3-phosphate dehydrogenase; amplicon: 255 bp, Gene ID: 14433).

### Statistical analyses

Statistical comparisons were operated using GraphPad Prism 9. Data were presented as mean ± s.e.m. The *t*-test was used for the analysis and comparison of the data between two groups, while the two-way ANOVA was used for the statistical analysis among multiple groups, and the multiple comparison was based on Sidak’s tests. Normal distribution for the *t*-test was verified. *P* < 0.05 was statistically different.

### Ethical statement

All experiments were carried out in conformity to the ARRIVE guidelines for the Care and Use of Laboratory Animals, and were approved by Animal Care and Ethics Committee of Hebei Medical University. All methods were performed in accordance with the relevant guidelines and regulations.

## Data Availability

All data generated or analyzed during this study are included in this published article. Data are available on request from the corresponding author.
